# Flow volume curve: A diagnostic tool in extrathoracic airway obstruction

**DOI:** 10.12669/pjms.36.4.2283

**Published:** 2020

**Authors:** Thamir Al-Khlaiwi

**Affiliations:** Thamir Al-Khlaiwi, PhD (USA). Assistant Professor, Department of Physiology, College of Medicine, King Saud University and King Saud University Medical City (KSUMC), Riyadh, Saudi Arabia

**Keywords:** Flow-volume curve, Fixed Extrathoracic Airway obstruction

## Abstract

The flow-volume loop (F/V-loop) is a presentation of inhalation and exhalation of air stream volume during inspiration and expiration. It demonstrates the obstructive, restrictive and mixed pattern lung pathology. Flow-volume loop has been extensively used for evaluating the severity, progression and resolution of various causes of upper-airway conditions.

## CASE REPORT

A 28-year-old male Saudi patient, height 160 cm; weight 85.3 kg, with a 2-month earlier history of road traffic accident was referred to King Abdulaziz University Hospital. Patient had a history of tracheostomy, managed with endotracheal intubation and mechanical ventilation for two weeks. Post hospital discharge, within two weeks, patient started exertional breathlessness associated with dyspnea.

**Fig.1:**
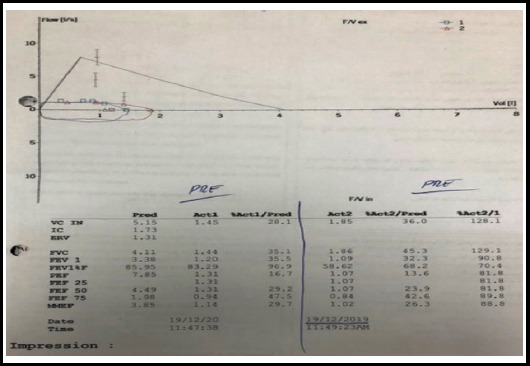
Flow volume loop in fixed upper airway obstruction.

Patient was referred for lung function testing. The complete blood picture and biochemistry tests were normal. The lung function test parameters were recorded in sitting position without bronchodilator challenge. Forced Vital Capacity (FVC), Forced Expiratory Volume in First Second (FEV1), Peak Expiratory Flow (PEF), Forced Expiratory Flow 50% (FEF-50%), Forced Expiratory Flow 75% (FEF-75%), and Maximum Mid Expiratory Flow (MMEF) were decreased, however, FEV1 /FVC ratio was within normal limit (Table-I). Both inspiratory and expiratory loop volume curve shows a blunt response, box pattern (Fig.1).

**Table-I:** Pulmonary function test of the patient.

Parameters	Predicted Range	Actual Results	Percentage
FVC (lit / sec)	4.11	1.44	35.1
FEV1 (Lit)	3.38	1.20	35.5
FEVI/FVC %	85.95	83.29	96.9
PEF (lit sec)	7.85	1.31	16.7
FEF-50% (lit / sec)	4.49	1.31	29.2
FEF-75% (lit / sec)	1.98	0.94	47.5
MMEF% (lit / sec)	3.85	1.14	29.7

## DISCUSSION

The F/V curve is a representation of air stream volume pattern during inspiration and expiration. It exhibits the obstructive, restrictive and mixed pattern pathological conditions. It is extensively used for evaluating the severity, progression and resolution of various causes of upper airway obstruction including post-surgical changes, before and after resection of airway tumors, correction of tracheal stenosis, abnormalities with neuromuscular diseases and vocal-cord paralysis^1^. The flow volume curves shape gives an evidence whether the curve is normal or abnormal and disease pattern is obstructive, restrictive or mixed ventilatory defects^1^,^2^.

The spirogram exhibits a configuration which reflects the underlying physiological and pathological state of the respiratory system. The principal clinical importance of the flow volume loop is to that it demonstrates whether the airflow is normal for a particular lung volume or not. The classic flow-volume loop shapes describe the obstructive and restrictive lung pathology as well as anatomical abnormalities 3. Trusting in spirometric test values without investigating and understanding the shape of the flow-volume loop may lead to inappropriate or misdiagnosis and mismanagement of respiratory diseases.^4^ Flow-volume loop has been extensively used for evaluating the severity, progression and resolution of various causes of upper-airway obstruction including post-surgical changes, before and after resection of airway tumors, correction of tracheal stenosis, abnormalities with neuromuscular diseases, parkinson disease and vocal-cord paralysis 5.

It has also been reported that an intra-thoracic tracheal obstruction such as tumor forced the outer upper air ways structures due to negative pressure created during inhalation, producing a normal shaped inspiratory component of F/V loop. While during exhalation phase the causing obstruction material is sucked in the trachea, producing flattened exhalation of F/V loop. To determine such situation, it is essential to know, the clinical history, loop volume curve, lung function test parameters and Empey index. The Empey index, calculated as FEV1 (ml/s) divided by the PEF (l/min)]. Empey index values more than 10 considered abnormal^6^. In this case, considering all these parameters, including Flow Volume curve shape, it was identified that patient has a fixed extrathoracic airway obstruction.
